# Development and Evaluation of the 40K Liquid SNP Array for Population Genetic Analysis in Golden Pompano (*Trachinotus ovatus*)

**DOI:** 10.3390/ijms27209263

**Published:** 2026-10-19

**Authors:** Baosuo Liu, Yayang Gao, Huayang Guo, Kecheng Zhu, Lin Xian, Nan Zhang, Tengfei Zhu, Dianchang Zhang

**Affiliations:** 1 Key Laboratory of South China Sea Fishery Resources Exploitation and Utilization, Ministry of Agriculture and Rural Affairs, South China Sea Fisheries Research Institute, Chinese Academy of Fishery Sciences, Guangzhou 510300, China; liubaosuo343@163.com (B.L.); southchinasfrigao@163.com (Y.G.); guohuayang198768@163.com (H.G.); zkc537@163.com (K.Z.); c-xianlin@genomics.cn (L.X.); 398730316@163.com (N.Z.); zhtfsuper@outlook.com (T.Z.); 2 Sanya Tropical Fisheries Research Institute, Sanya 572018, China; 3 Guangdong Engineering Research Center of Key Technologies and Equipment R&D on Modern Marine Ranching, Guangzhou 510300, China; 4 Guangdong Provincial Engineer Technology Research Center of Marine Biological Seed Industry, Guangzhou 510300, China

**Keywords:** *Trachinotus ovatus*, liquid SNP array, population genetics, genetic diversity, genomic breeding

## Abstract

Single nucleotide polymorphism (SNP) arrays provide efficient and scalable platforms for genetic diversity assessment, population genomic analysis, and molecular breeding in aquaculture species. However, a high-throughput SNP genotyping platform specifically developed for golden pompano (*Trachinotus ovatus*), an economically important marine aquaculture fish, remains limited. This study aimed to develop and evaluate a liquid SNP array for *T. ovatus* and assess its applicability in population genetic analyses. Genome-wide SNP resources were generated from 200 individuals representing 10 cultured populations of *T. ovatus*. Following SNP discovery, quality filtering, probe evaluation, and marker optimization, a 40K liquid SNP array, designated Changxin-I, was constructed. The performance of Changxin-I was evaluated using an independent validation cohort of 144 individuals from five cultured populations with both WGS-derived and array-derived genotype data available. The array exhibited high genotyping performance, with an average SNP detection rate of 99.94% and a genotype concordance rate of 96.23% with WGS data. Genetic diversity and population structure analyses were performed using Changxin-I genotype data, and the inferred population patterns were compared with those obtained from whole-genome resequencing (WGS) data from the same individuals. A total of 40,476 SNPs were selected for the final Changxin-I array, including 40,442 genome-wide background markers and 34 trait-associated SNPs. The validation results showed high genotyping quality, with an average mapping rate of 98.87% and a mean SNP detection rate of 99.94%. Most SNP markers (99.93%) exhibited minor allele frequencies (MAF) greater than 0.10. Changxin-I successfully revealed genetic diversity and population differentiation among cultured populations, and the population structure patterns were consistent with those obtained from WGS-based analyses. Changxin-I represents a reliable and efficient genomic tool for large-scale SNP genotyping in golden pompano. This array provides a practical platform for population genetic studies, germplasm characterization, and future genomic-assisted breeding applications in *T. ovatus*.

## 1. Introduction

The rapid development of aquaculture breeding programs has increased the demand for genomic tools that can accurately characterize genetic variation and facilitate genetic improvement. Molecular markers have therefore become essential resources for assessing germplasm diversity, resolving population structure, and supporting marker-assisted and genomic selection strategies in cultured species [1,2,3]. Among available marker systems, single nucleotide polymorphisms (SNPs) have become particularly valuable owing to their high abundance throughout the genome, stable inheritance patterns, biallelic nature, and compatibility with automated high-throughput genotyping platforms. Genome-wide SNP markers provide substantially higher resolution for evaluating genetic relationships and population structure than many traditional marker systems and are also suitable for identifying genomic regions and candidate genes associated with economically important traits [4]. Consequently, SNP-based genotyping has become an important component of marker-assisted selection (MAS) and genomic selection (GS), which are increasingly being incorporated into genetic improvement programs for aquaculture species [4,5].

The effectiveness of modern genomic breeding depends strongly on the availability of reliable and cost-effective high-throughput genotyping platforms [6]. Whole-genome sequencing (WGS) can provide comprehensive information on genome-wide genetic variation and has greatly accelerated SNP discovery and population genomic studies in aquaculture [7]. However, its routine application to large breeding populations is still constrained by sequencing costs, large data volumes, and the computational resources required for read processing, variant calling, filtering, and downstream analyses [8]. In contrast, SNP arrays provide a practical solution by targeting predefined informative markers, enabling efficient and standardized genotyping for population analyses, genetic mapping, genome-wide association studies, and genomic prediction. Conventional solid-phase SNP arrays have been successfully developed for several economically important aquatic species, including Rainbow trout (*Oncorhynchus mykiss*) [9], Nile Tilapia (*Oreochromis niloticus*) [10], and other major cultured fishes. These platforms have demonstrated the value of genome-wide SNP genotyping in genetic mapping, population analysis, GWAS, and genomic prediction.

Liquid-phase capture-based SNP genotyping has emerged as a flexible alternative to conventional solid-phase arrays. Genotyping-by-target sequencing (GBTS) uses specifically designed probes to enrich predefined genomic regions, followed by high-throughput sequencing and genotype calling [11]. Because markers can be selected according to genome-wide distribution, polymorphism, functional relevance, and breeding objectives, liquid SNP arrays offer considerable flexibility in panel design and modification [12]. They also reduce the amount of sequencing required relative to WGS while retaining the advantages of high-throughput and scalable SNP genotyping [13]. These characteristics are particularly advantageous for aquaculture species, for which breeding populations may contain substantial genetic diversity and species-specific commercial arrays are often unavailable [14]. In recent years, liquid SNP arrays have been developed for several aquatic species, including leopard coral grouper (*Plectropomus leopardus*) [15], blunt snout bream (*Megalobrama amblycephala*) [16], Chinese mitten crab (*Eriocheir sinensis*) [17], spotted sea bass (*Lateolabrax maculatus*) [18], and silver carp (*Hypophthalmichthys molitrix*) [19]. These studies have demonstrated the potential of liquid SNP arrays for genetic diversity assessment, population structure analysis, germplasm identification, GWAS, disease-resistance studies, and genomic breeding. The increasing application of these platforms indicates that a moderately sized panel containing highly informative and well-distributed SNPs can provide an efficient solution for large-scale genotyping in aquaculture breeding populations.

The golden pompano, *Trachinotus ovatus*, is an economically important marine aquaculture species in southern China. According to the China Fishery Statistical Yearbook 2025, its annual aquaculture production reached 305,552 tons, making it the most extensively produced marine finfish species in China [20]. It is highly valued for its rapid growth, superior flesh quality, and high commercial value. With the expansion of artificial propagation and intensive aquaculture, genetic management of cultured populations has become increasingly important. Long-term use of limited broodstock, exchange of seed among farms, and repeated artificial propagation may alter the genetic composition of cultured stocks and increase the risk of reduced genetic diversity or genetic homogenization [21,22]. Therefore, accurate characterization of germplasm resources and the establishment of efficient genomic tools are important for broodstock management and future genetic improvement of *T. ovatus*. Previous studies have already established a foundation for molecular breeding in this species. Microsatellite markers have been used to characterize genetic diversity and population structure among cultured *T. ovatus* populations, and genome-wide approaches have identified SNPs associated with economically important traits [23]. For example, GWAS has revealed candidate SNPs and genes associated with hypoxia tolerance [24]. These advances provide valuable genomic resources for marker selection and molecular breeding. Nevertheless, the continued expansion of golden pompano breeding programs creates a practical demand for a high-throughput liquid SNP genotyping platform that combines genome-wide marker coverage with high polymorphism and incorporates markers associated with economically important traits.

In this study, we developed Changxin-I, a 40K liquid SNP array specifically designed for *T. ovatus*. Genome-wide SNP discovery was conducted using 200 individuals representing 10 cultured populations in southern China. Candidate markers were selected based on genome-wide distribution, polymorphism, probe designability, and functional relevance, including SNPs associated with growth, hypoxia tolerance, *Cryptocaryon irritans* resistance, and sex determination. The performance of Changxin-I was subsequently evaluated in an independent validation panel, and its effectiveness for population genetic analysis was further assessed through comparison with WGS-derived genomic variation. This study establishes a scalable genomic platform for genetic diversity assessment, germplasm characterization, and genome-assisted breeding in *T. ovatus*.

## 2. Results

### 2.1. Development and Genomic Characteristics of the Changxin-I 40K Liquid SNP Array

Genome-wide SNP discovery in the 200-individual development panel initially identified 13,982,173 SNPs. After excluding low-depth variants and filtering based on genotype missing rate (<10%) and minor allele frequency (MAF ≥ 0.10), 1,515,528 candidate SNPs were retained. Following probe-design evaluation, loci with successfully designed probes were selected according to genome-wide distribution criteria, resulting in 45,454 SNPs for experimental testing. Based on probe-capture stability and genotyping performance, 40,476 SNPs were retained as the final marker set incorporated into Changxin-I.

**Figure 1:**
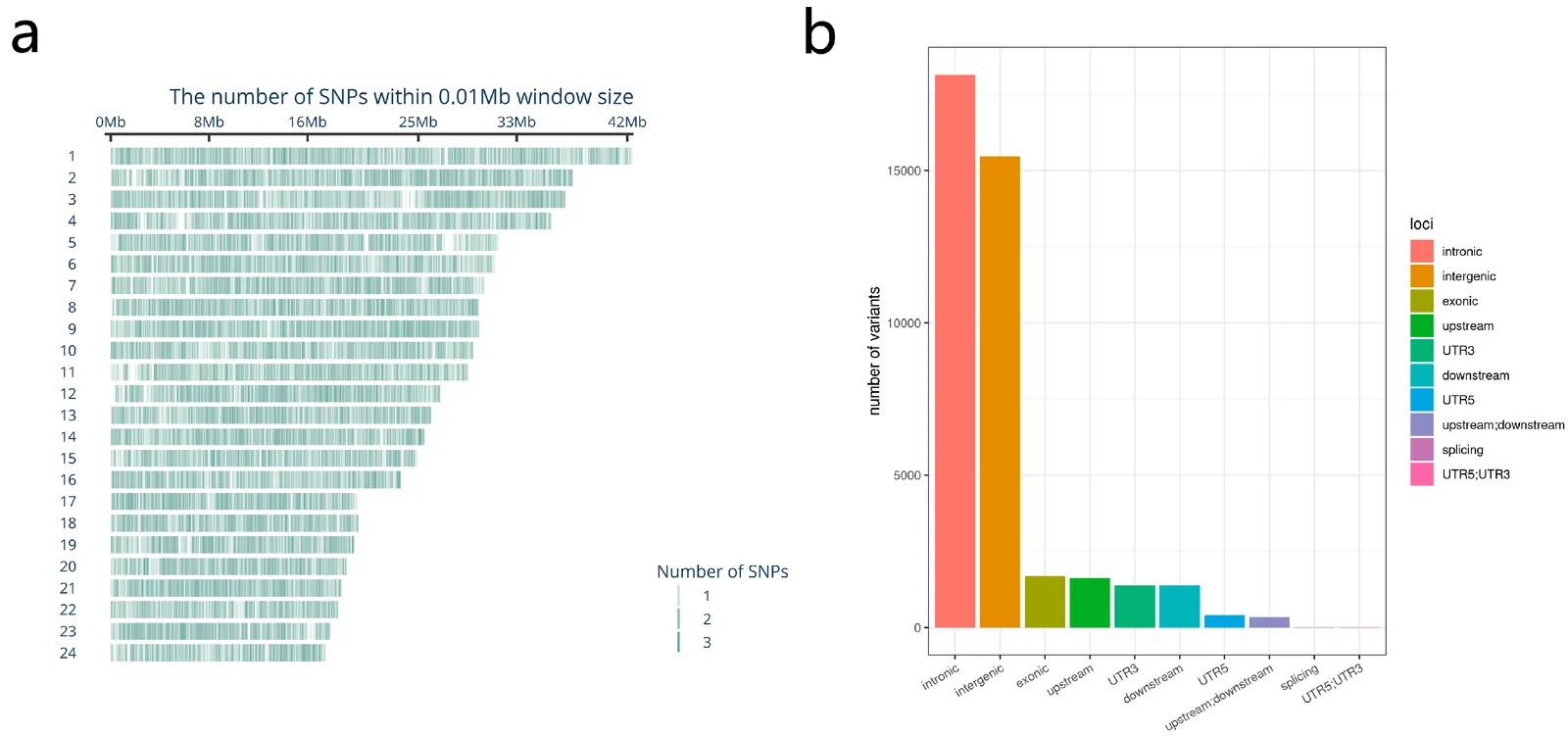
Genomic characteristics and polymorphism of SNPs in the Changxin-I 40K liquid SNP array. (**a**) Genome-wide distribution of SNPs across the 24 chromosomes of *T. ovatus*, with SNP density calculated in 10 kb windows. (**b**) Functional annotation of SNPs across different genomic regions.

The 40,476 SNPs were distributed across all 24 chromosomes of *T. ovatus* (Figure 1a). Mean distances between adjacent SNPs across individual chromosomes ranged from approximately 13.66 to 17.56 kb, indicating broadly comparable marker spacing among chromosomes. Functional annotation showed that 18,142 SNPs (44.82%) were located in intronic regions and 15,470 (38.22%) in intergenic regions, together accounting for 83.04% of the Changxin-I markers (Figure 1b). The remaining SNPs were distributed across exonic, upstream, downstream, untranslated, and splice-related regions.

### 2.2. Genotyping Performance and Polymorphism of the Changxin-I Array

An independent validation cohort comprising 144 individuals from five cultured populations (ZJ, BH, SY, SZ, and LS) was used to evaluate the genotyping performance of Changxin-I. Whole-genome resequencing data and Changxin-I genotyping data were obtained from the same individuals, allowing direct comparison between array-derived and WGS-derived genotypes. A total of 228.74 Gb of raw sequencing data were generated, corresponding to an average of 1.59 Gb per individual. After quality filtering, 215.71 Gb of clean data were retained, with a mean effective rate of 94.30%. The mean Q20 and Q30 values were 99.21% and 97.29%, respectively. Clean reads were aligned to the *T. ovatus* reference genome with a mean mapping rate of 98.87%.

**Figure 2:**
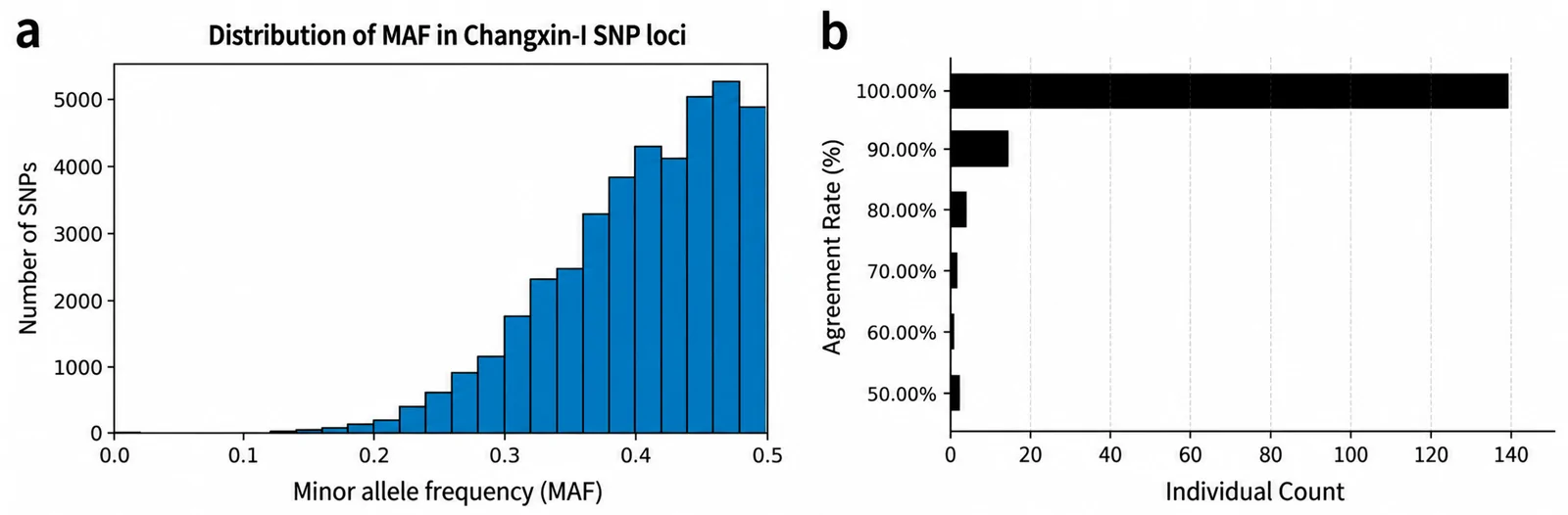
(**a**) MAF distribution calculated from Changxin-I genotypes of 144 individuals from the independent validation cohort. (**b**) Technical performance evaluation of Changxin-I liquid SNP array.

SNP detection rates ranged from 99.43% to 99.97% among individuals, with a mean of 99.941%. All 144 individuals showed SNP detection rates above 90%, with an average detection rate of 99.94%. Among the 40,464 loci with callable genotypes, 40,437 SNPs (99.93%) had MAF values > 0.10, and 57.91% had MAF values ≥ 0.40 (Figure 2a). After downstream quality control, among the 40,476 SNPs, 78 loci were removed during quality control due to missing genotype rate, low MAF, or deviation from HWE, 40,398 SNPs were retained for subsequent population genetic analyses. The average SNP detection rate across all loci was 99.94%, and the genotype concordance rate between Changxin-I and WGS-derived genotypes reached 96.23%. (Figure 2b). Although most individuals showed high concordance, a small number of individuals exhibited relatively lower concordance values. These differences may result from platform-specific genotype calling procedures, particularly for loci with ambiguous allele assignment or limited read support during WGS-based genotype inference. Given the overall high concordance across the validation cohort, these individual-level differences are unlikely to represent systematic genotyping errors.

### 2.3. Genetic Diversity and Differentiation Among Populations

To evaluate the applicability of Changxin-I for population genetic analysis, genetic diversity was assessed in the five cultured populations using the 40,398 high-quality SNPs retained after quality control (Table 1). Observed heterozygosity (*H*o) ranged from 0.4604 in BH to 0.4776 in SZ, whereas expected heterozygosity (*H*e) ranged from 0.4529 in SY to 0.4705 in BH. Polymorphism information content (PIC) varied from 0.3439 in SY to 0.3545 in BH, and nucleotide diversity (π) ranged from 5.7114 × 10^−5^ in SY to 5.9318 × 10^−5^ in BH. The population rankings based on *H*e, PIC, and π were identical (BH > LS > ZJ > SZ > SY), whereas Ho showed a different pattern, with the highest value in SZ and the lowest in BH.

Hardy–Weinberg equilibrium (HWE) tests revealed only limited nominal departures among the five populations. At *p* < 0.05, the proportion of SNPs deviating from HWE ranged from 2.66% in ZJ to 4.04% in SY. After Benjamini–Hochberg false discovery rate correction, no SNP remained significantly deviated from HWE in any population.

Population differentiation was further examined using pairwise *F*_ST_ and Nei’s genetic distances (Table 2). Pairwise *F*_ST_ values ranged from 0.0090 to 0.0685. Seven of the ten population pairs had *F*_ST_ values below 0.05, whereas values above 0.05 were observed for ZJ–SY (0.0547), SY–LS (0.0564), and SY–SZ (0.0685). The lowest *F*_ST_ was detected between ZJ and BH (0.0090), while the highest occurred between SY and SZ (0.0685). Nei’s genetic distances ranged from 0.0227 to 0.0764 and showed the same extrema, with the shortest distance between ZJ and BH and the largest between SY and SZ. Permutation tests based on 1999 randomizations revealed statistically significant genetic differentiation for all ten population pairs after Benjamini–Hochberg FDR correction (all empirical *p* = 0.0005; FDR-adjusted q = 0.0005). Despite their statistical significance, the magnitudes of pairwise *F*_ST_ varied among populations, with the lowest differentiation observed between ZJ and BH and the highest between SY and SZ.

### 2.4. Application of Changxin-I in Population Structure Analysis

Population structure was evaluated using PCA, phylogenetic reconstruction, and ADMIXTURE analyses based on both Changxin-I SNP array and WGS datasets. A total of 4025.42 Gb of raw WGS data were generated, of which 4004.85 Gb were retained after quality filtering. The clean reads had mean Q20 and Q30 values of 96.54% and 89.82%, respectively, with an average mapping rate of 98.79%, sequencing depth of 42.76×, and genome coverage of 98.25%. After variant quality control, 795,377 high-quality biallelic SNPs were retained, with an overall genotype call rate of 99.88%. The array and WGS datasets were subsequently analyzed separately and compared using PCA, phylogenetic analysis, and ADMIXTURE.

**Figure 3:**
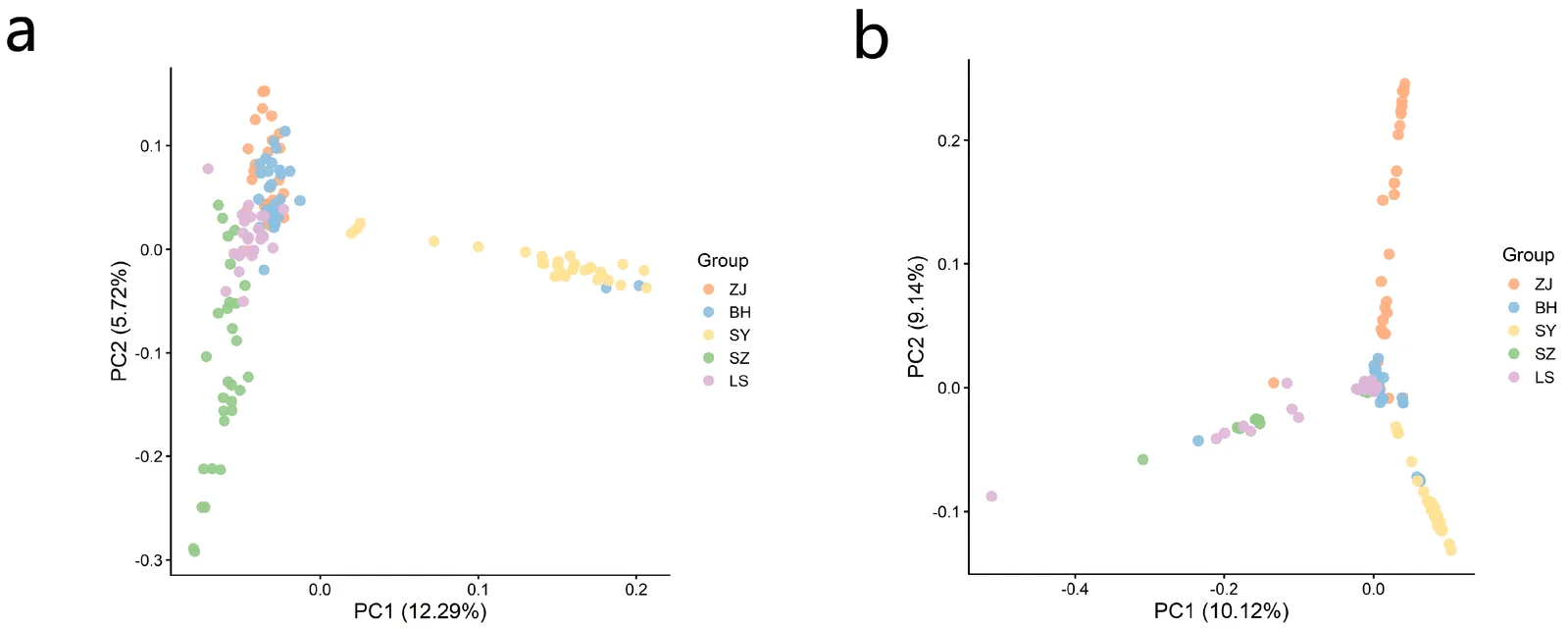
Principal component analysis (PCA) of 144 *T. ovatus* individuals from five populations based on Changxin-I SNP array and whole-genome resequencing (WGS) datasets. (**a**) PCA based on Changxin-I SNP genotypes. (**b**) PCA based on WGS-derived SNP genotypes.

PCA based on the Changxin-I SNPs showed that PC1 and PC2 explained 12.29% and 5.72% of the genetic variation, respectively (Figure 3a). SY was clearly separated from the other populations, whereas ZJ and BH showed substantial overlap. SZ showed a relatively broad distribution, while LS was mainly distributed close to ZJ, BH, and SZ. PCA based on the WGS SNPs showed that PC1 and PC2 explained 10.12% and 9.14% of the variation, respectively (Figure 3b). ZJ and SY were relatively well separated, SZ and LS showed a close distribution with partial overlap, and BH overlapped with several populations.

**Figure 4:**
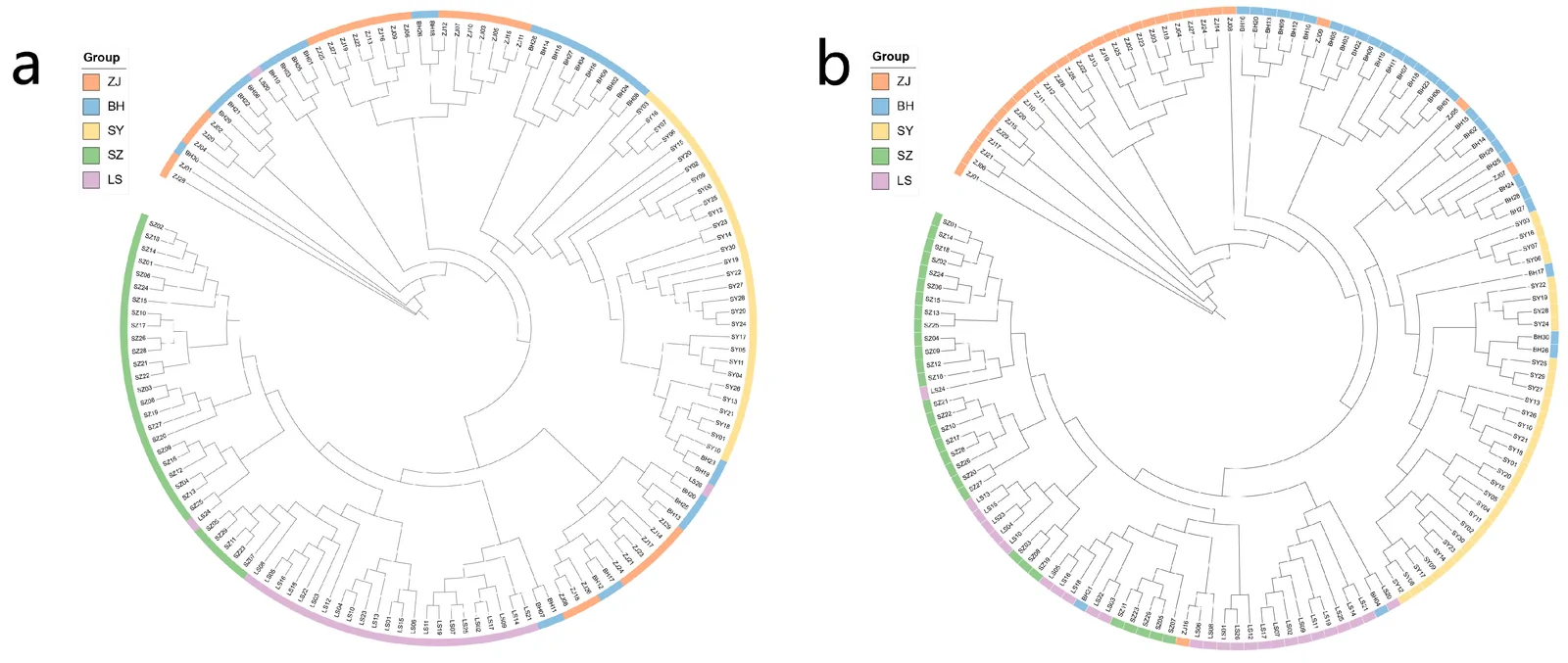
Maximum-likelihood phylogenetic trees of 144 *T. ovatus* individuals from five populations inferred from Changxin-I SNP array and whole-genome resequencing (WGS) datasets. (**a**) Phylogenetic tree constructed using Changxin-I SNP genotypes. (**b**) Phylogenetic tree constructed using WGS-derived SNP genotypes.

The phylogenetic tree based on the Changxin-I SNPs showed that individuals from the same population generally clustered in neighboring branches (Figure 4a). Most SY individuals formed a relatively concentrated cluster, ZJ and BH were mainly located in neighboring branches, and SZ and LS showed partial intermingling. The WGS-based phylogenetic tree showed a similar overall relationship among the five populations (Figure 4b), particularly the clustering of most SY individuals and the close relationship between SZ and LS. ZJ and BH also showed partial intermingling in neighboring branches.

**Figure 5:**
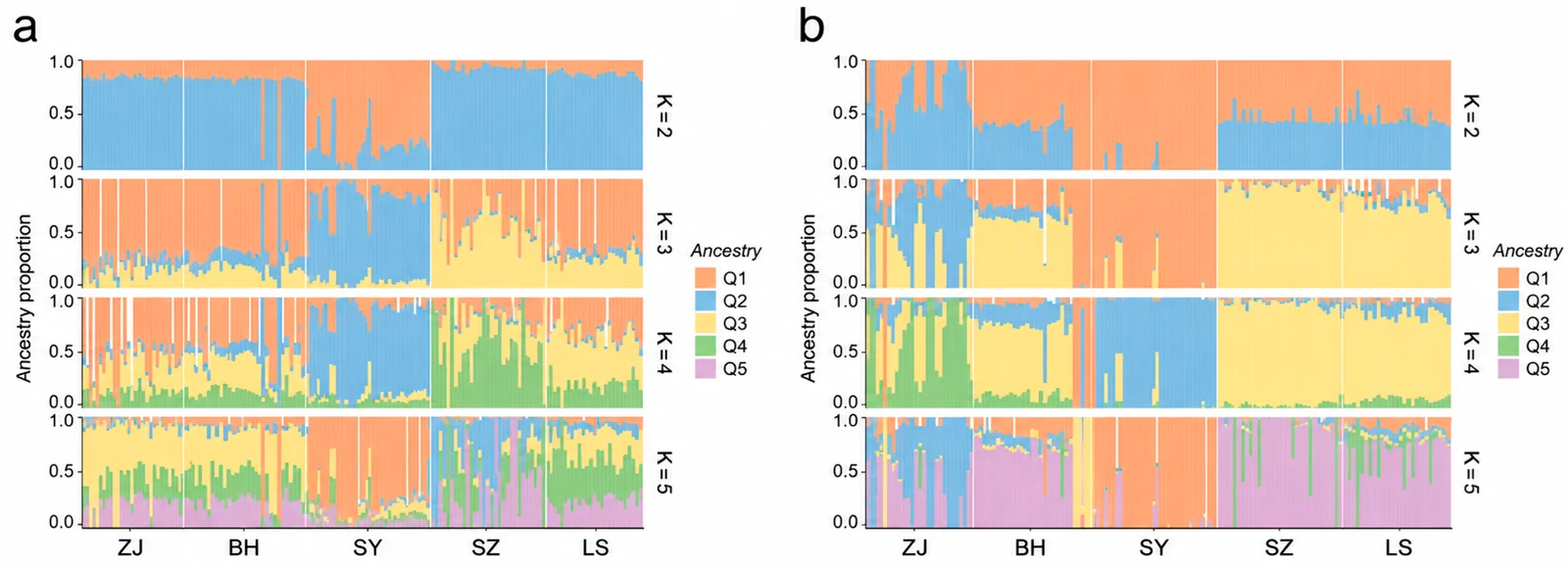
ADMIXTURE analysis of 144 *T. ovatus* individuals from five populations based on Changxin-I SNP array and whole-genome resequencing (WGS) datasets. (**a**) ADMIXTURE analysis based on Changxin-I SNP genotypes. (**b**) ADMIXTURE analysis based on WGS-derived SNP genotypes for K = 2–5. Each vertical bar represents one individual, and different colors represent inferred genetic clusters.

ADMIXTURE analysis based on the Changxin-I SNPs showed increasing population subdivision from K = 2 to K = 5 (Figure 5a). At K = 2, SY was already differentiated from the other populations. At K = 3, the differentiation of SY remained evident, while ZJ and BH showed similar population structure and SZ and LS also exhibited a relatively close relationship. Further subdivision within populations was observed at K = 4 and K = 5. The WGS-based ADMIXTURE analysis showed a comparable overall pattern (Figure 5b), with SY remaining relatively distinct and SZ and LS showing a close relationship across multiple K values. Overall, the major population relationships revealed by the array were comparable to those obtained from WGS, although differences were observed in the detailed clustering patterns. These differences may result from variations in marker density and SNP selection strategies between the two datasets. WGS captures a much larger number of genomic variants, whereas Changxin-I consists of a predefined set of informative SNP markers optimized for stable and efficient genotyping. These results support the applicability of Changxin-I for capturing the major population genetic patterns of *T. ovatus*.

## 3. Discussion

Liquid-based SNP array platforms have emerged as powerful tools for large-scale genomic analysis due to their advantages in high-throughput genotyping and sensitive marker detection. These technologies have increasingly contributed to aquaculture applications, including selective breeding programs and the assessment of genetic diversity and germplasm resources. To date, liquid SNP arrays have been successfully developed and utilized in various economically important aquatic organisms. For instance, a 7K SNP array was employed to investigate genetic differentiation between wild and cultured Atlantic salmon (*Salmo salar* L.) [25]. In European seabass (*Dicentrarchus labrax*) and gilthead seabream (*Sparus aurata*), a 60K SNP array provided effective discrimination of population genetic patterns [26]. Moreover, the application of a 55K SNP array in large yellow croaker (*Larimichthys crocea*) achieved reliable genotyping performance and high repeatability, facilitating studies on genetic improvement and germplasm evaluation in aquaculture species [8,12]. In this study, we developed and evaluated a 40K liquid SNP array for *T. ovatus* using the genotyping-by-target sequencing (GBTS) approach. The broad genome-wide distribution of SNP markers and high SNP detection rate demonstrated the technical performance of Changxin-I. Furthermore, its ability to identify major patterns of genetic differentiation and population structure among cultured populations supports its applicability to population genetic analysis and provides a useful genomic resource for future germplasm evaluation.

Whole-genome resequencing and genotyping-by-sequencing technologies enable broad coverage of genome-wide loci and have become important tools for obtaining comprehensive genetic information [7]. In most species, including lungfish (*Lepidosiren paradoxa*) [27], and krill (*Euphausia superba*) [28], non-coding regions occupy a large proportion of the genome. Although such comprehensive sequencing approaches can detect all variant sites, not all SNPs are equally valuable in genetic breeding. Low-frequency variants, loci in strong linkage disequilibrium, and redundant SNPs often contribute minimally to trait variation and may not provide biologically meaningful information [29]. In contrast, liquid-phase SNP chip technology offers higher target specificity during design and development, effectively reducing the interference of redundant markers [11]. By screening for high-quality, highly polymorphic SNPs with significant effects on specific traits or functions [30]. The potential of this approach has been demonstrated in multiple aquaculture species. For example, Zhou et al. successfully applied a 50 K SNP chip in Japanese flounder (*Paralichthys olivaceus*) for genomic selection targeting *Edwardsiella tarda* resistance [31]. Yáñez et al. used a 50 K SNP panel for high-quality SNP analysis across three Nile tilapia populations [32], while Ye et al. developed a 40 K liquid-phase chip for genomic selection of growth traits in mud crab (*S. paramamosain*) [13]. These cases highlight that liquid-phase SNP chips provide a targeted and scalable platform for efficient genotyping in breeding applications, underscoring their high potential and value in targeted breeding applications.

The high proportion of polymorphic SNPs observed in the Changxin-I validation populations suggests that the marker selection strategy effectively retained informative loci, supporting the applicability of the array in population genetic analyses. For instance, the liquid-phase SNP array developed for *S. paramamosain* contained 92.70% of SNPs with MAF > 0.10 [13], while SNP arrays developed for *Piaractus brachypomus* and *Piaractus mesopotamicus* contained approximately 70% of SNPs with MAF > 0.05 [33]. However, differences in MAF thresholds and sampled populations should be considered when comparing marker polymorphism across studies.

The predominance of SNP markers in non-coding genomic regions, particularly intronic and intergenic regions, is consistent with the genomic distribution patterns reported for liquid-phase SNP arrays developed for sea cucumber (*Apostichopus japonicus*) and Pacific abalone (*Haliotis discus hannai*) [34,35]. The distribution of SNP markers across different genomic regions, together with their genome-wide chromosomal coverage, supports the potential applicability of Changxin-I in downstream analyses, including genetic diversity assessment and population structure investigation.

The Changxin-I SNP array provides a useful tool for assessing genetic diversity in cultured *T. ovatus* populations. π and heterozygosity are commonly used parameters for evaluating population genetic variation and have been widely applied in aquatic genetic resource studies [36,37]. *H*o represents the proportion of heterozygous loci within a population and provides an indication of genetic variation [38]. The relatively comparable *H*o and *H*e values observed among the five populations suggest broadly similar levels of heterozygosity at the investigated SNP loci. Luo et al. evaluated wild and cultured populations of orange-spotted grouper (*Epinephelus coioides*) and reported relatively high genetic diversity [39]. Dong et al. investigated nine grouper species using microsatellite markers and found moderate levels of genetic variation, with *H*o values ranging from 0.4615 to 0.6239 and *H*e values ranging from 0.3510 to 0.4754 [40]. Nucleotide diversity has also been widely used to investigate genetic variation in marine fish populations [41]. The π estimates obtained in the present study were of the same order of magnitude as those reported for six spotted sea bass populations analyzed using a 40K liquid-phase SNP array [42]. However, differences in marker selection and sampling should be considered when comparing genetic diversity estimates across studies. According to commonly used classification criteria, PIC values between 0.25 and 0.50 indicate moderate polymorphism [43]. The moderate PIC values observed in the five golden pompano populations suggest that the selected markers provide useful polymorphic information for population genetic analyses. Similar levels of genetic diversity have also been reported in cultured aquatic populations [42]. Furthermore, the absence of significant deviations from Hardy–Weinberg equilibrium after FDR correction suggests that no strong departures were detected at the assayed loci, although this does not establish long-term genetic stability. Overall, these findings support the applicability of Changxin-I for characterizing genetic diversity in cultured *T. ovatus* populations.

To further understand the genetic relationships among the five cultured populations, genetic differentiation was evaluated using pairwise *F*_ST_ and genetic distance measures [40]. Although all pairwise comparisons showed statistically significant differentiation after permutation testing and FDR correction, the generally low to moderate *F*_ST_ values indicated that the magnitude of differentiation was relatively limited. The SY population exhibited relatively higher genetic differentiation from the other populations, particularly SZ, suggesting a comparatively distinct genetic composition. In contrast, relatively low levels of genetic differentiation were observed among ZJ, BH, SZ, and LS, suggesting that these populations maintained relatively close genetic relationships. Similar patterns of genetic differentiation have also been discussed in population genetic studies of the speckled blue grouper (*Epinephelus cyanopodus*) [44].

Population genetic structure analyses based on Changxin-I SNP genotypes provided complementary insights into the genetic relationships among the five cultured *T. ovatus* populations. The broadly consistent patterns obtained from PCA, phylogenetic analysis, and ADMIXTURE supported the *F*_ST_-based interpretation that SY had a relatively distinct genetic composition, whereas ZJ and BH were genetically more similar., although these patterns do not provide direct evidence of historical gene flow or shared broodstock origins. Taken together, these findings suggest that Changxin-I captures the major features of genetic structure among the sampled cultured populations, while further demographic or pedigree information would be required to clarify the processes underlying these patterns.

Comparison of population genetic structure inferred from Changxin-I and WGS data from the same 144 individuals provided further evidence of the applicability of the SNP array for population genetic analyses. Despite differences in the number and composition of SNP markers, the two datasets revealed broadly similar major population relationships. The observed differences in detailed clustering patterns may result from differences in marker density and SNP selection strategy. WGS provides a larger number of genome-wide variants and may capture additional signals associated with fine-scale differentiation, in addition, the SNP ascertainment process may contribute to differences between Changxin-I and WGS datasets. During array development, SNP markers were selected based on multiple criteria, including minor allele frequency, genome-wide distribution, probe designability, and genotyping stability. These procedures improve the reliability and reproducibility of genotyping but may reduce the representation of rare variants or population-specific loci that can contribute to subtle genetic differentiation. Therefore, the observed differences in fine-scale clustering patterns are expected and reflect the distinct characteristics of targeted SNP arrays and whole-genome sequencing approaches., whereas Changxin-I represents a predefined marker panel optimized for stable genotyping and practical population analyses, suggesting that Changxin-I retained sufficient genetic information to characterize the principal patterns of population structure among the sampled populations. Nevertheless, differences in the detailed clustering patterns indicate that the two marker datasets are not entirely equivalent. A similar validation approach was adopted in the development of the 50K liquid-phase Fangxin-I SNP array for *M. amblycephala*, in which phylogenetic relationships inferred from array-derived genotypes were compared with those obtained from whole-genome resequencing data [16]. The technical reliability of the 40K liquid SNP array was supported by comparison between Changxin-I genotype calls and corresponding WGS-derived genotypes from individuals in an independent validation cohort. The high SNP detection rate and genotype concordance with resequencing-derived genotypes indicate that the selected markers and probe design strategy provided accurate genotype calling.

The development of Changxin-I provides an efficient genomic resource for accelerating genetic studies and breeding applications in *T. ovatus*. Several limitations should be considered. Although SNP selection was based on informative polymorphic loci and may not represent all genomic variants, trait-associated markers included in the panel should be further evaluated through independent association studies before practical application in marker-assisted selection. With the integration of additional phenotypic datasets and breeding populations, the array has strong potential for applications in GWAS, genomic selection, and marker-assisted breeding, thereby contributing to the sustainable genetic improvement of golden pompano aquaculture.

## 4. Materials and Methods

### 4.1. Sample Collection

A total of 200 *T. ovatus* individuals from 10 major aquaculture populations in southern China were used for SNP discovery and array development, with 20 individuals sampled from each population. The populations originated from Zhanjiang (*n* = 20), Shenzhen (*n* = 20), Yangjiang (*n* = 20), and Zhuhai (*n* = 20) in Guangdong Province; Beihai (*n* = 20) and Qinzhou (*n* = 20) in Guangxi Zhuang Autonomous Region; and Changjiang (*n* = 20), Sanya (*n* = 20), Lingshui (*n* = 20), and Lingao (*n* = 20) in Hainan Province. These individuals constituted the array-development panel and were used exclusively for genome-wide SNP discovery and marker selection. All individuals were collected from commercial hatcheries or farms, and fin clips were sampled for genomic DNA extraction. Individuals were randomly selected to minimize sampling bias.

An independent validation cohort comprising 144 individuals from five cultured populations (ZJ, BH, SY, SZ, and LS) was used for Changxin-I evaluation. Both WGS and Changxin-I genotype datasets were available for these individuals, enabling direct comparison between WGS-based and array-based genotyping results. The collection locations and sample sizes of these 144 individuals are summarized in Table 3. These individuals were distinct from the 200 individuals used for array development. Corresponding WGS data were available for these 144 individuals and were used as the reference dataset for evaluating population genetic patterns inferred from Changxin-I. Caudal fin samples used for WGS were preserved in 75% ethanol at −20 °C prior to DNA extraction.

All procedures involving experimental animals were conducted in accordance with the guidelines of the Animal Care and Use Committee of the South China Sea Fisheries Research Institute, Chinese Academy of Fishery Sciences (approval no. SCSFRI96-241).

### 4.2. SNP Discovery and Candidate Marker Selection

Genome-wide SNP data generated from the 200-individual array-development panel were used as the initial marker source for Changxin-I development. Genotypes supported by sequencing depths < 5× were excluded prior to marker screening. Candidate SNPs were subsequently filtered using a genotype missing rate <10% and a minor allele frequency (MAF) ≥ 0.10. The 1,515,528 retained SNPs were evaluated for probe design feasibility. Loci with successfully designed probes were subsequently selected according to genome-wide distribution criteria to achieve broad and relatively even marker coverage across the 24 chromosomes of *T. ovatus*. A total of 45,454 SNPs were selected for experimental testing. Following experimental evaluation of the 45,454 candidate loci, SNPs showing stable probe capture performance were retained, resulting in a final marker set of 40,476 SNPs.

In addition to genome-wide background markers, previously identified SNPs associated with economically important traits, including growth, hypoxia tolerance, resistance to Cryptocaryon irritans, and sex, were included as trait-associated candidate markers (Table 4).

### 4.3. Probe Design, Experimental Testing, and Assembly of the Changxin-I SNP Array

Flanking sequences surrounding each candidate SNP were evaluated for probe designability. Probe evaluation considered sequence specificity, sequence complexity, GC content, and potential cross-hybridization. A 110 bp probe was designed for each candidate locus, and probes with GC contents outside the range of 30–70% or with more than five highly similar fragments in the reference genome were excluded. Candidate SNPs passing probe evaluation were prioritized according to their genomic positions to maintain an approximately even distribution of markers across the genome.

Candidate probes were synthesized using the GenoBaits^®^ GBTS platform (MolBreeding Biotechnology Co., Ltd., China) [45]. Probe performance was evaluated based on capture efficiency and genotype recovery rate using representative DNA libraries. Individual loci were evaluated according to probe-capture stability and the ability to generate reliable genotype calls across test samples. Loci exhibiting unstable capture or inadequate genotype detection were excluded during panel optimization. The retained SNPs were subsequently assembled into the final Changxin-I 40K liquid SNP array.

Functional annotation of the Changxin-I SNPs was performed using ANNOVAR (version 2020 Jun08) [46] based on the genomic positions and gene annotation of the *T. ovatus* reference genome. SNP distribution across the 24 chromosomes was summarized from genomic coordinates, and marker density was calculated using 10 kb windows.

### 4.4. Changxin-I Genotyping and Genotype Calling

Genomic DNA libraries were prepared following the manufacturer’s protocol for the GBTS platform. Target enrichment was performed using the Changxin-I probe panel, followed by high-throughput sequencing. Sequencing reads were aligned to the T. ovatus reference genome using BWA-MEM, and genotype calling was performed using the GATK Best Practices workflow.

Raw reads were filtered using fastp v0.20.0 [47] with the parameters -n 10 -q 20 -u 40. Adapter sequences were removed, read pairs containing more than 10 ambiguous bases were discarded, and read pairs were removed when bases with Phred quality scores ≤ 20 accounted for more than 40% of the read length.

Clean reads were aligned to the *T. ovatus* reference genome using the BWA-MEM algorithm [48]. Variant detection was performed following the Genome Analysis Toolkit (GATK) Best Practices workflow [49,50], target SNP genotypes were extracted using in-house Python v3.8.10 scripts. The project report explicitly identifies BWA-MEM for read alignment and GATK-based variant detection. For genotype assignment, SNP genotypes with read depth < 5× were recorded as missing. For heterozygous genotypes, each allele was required to be supported by at least four reads; otherwise, the genotype was also treated as missing.

For each individual, the SNP detection rate was calculated as the number of SNPs with valid genotype calls divided by the total number of 40,476 target SNPs. MAF was recalculated from the genotypes of the 144 validation individuals using only non-missing genotypes. SNP quality control for subsequent population analyses was performed using PLINK v1.9 [51].

### 4.5. Evaluation of Changxin-I Genotyping Performance

The performance of Changxin-I was assessed using the 144 validation individuals based on SNP call rate, genotype distribution, allele frequency spectrum, and concordance with resequencing-derived genotypes.

MAF was recalculated directly from the Changxin-I genotypes of the 144 validation individuals. For each SNP, allele frequencies were calculated using only non-missing genotypes, and MAF was defined as the frequency of the less common allele. Loci without any callable genotype among the 144 individuals were excluded from MAF calculation. The distribution of MAF was subsequently used to evaluate the polymorphism of the SNP panel.

Before population genetic analyses, SNP genotype data were subjected to quality control using PLINK. SNPs with excessive missing genotypes were removed, together with loci with low MAF or significant deviation from Hardy–Weinberg equilibrium (HWE). The filtering parameters were --mind 0.1 --geno 0.05 --maf 0.05 --hwe 0.000001. Linkage disequilibrium pruning was performed using --indep-pairwise 50 5 0.5 when required for population structure analyses. SNPs passing quality control were retained for subsequent genetic diversity, population differentiation, and population structure analyses.

Following array construction, the genotyping performance of Changxin-I was evaluated using 144 individuals with both WGS-derived genotypes and array-generated genotypes available. Genotype concordance between the two datasets was calculated to assess array accuracy.

### 4.6. Whole-Genome Resequencing and Variant Calling

Resequencing-derived genotypes were used as the reference dataset for evaluating the accuracy of Changxin-I genotype calls. Genomic DNA from the validation individuals was subjected to library construction and WGS. Qualified DNA was randomly fragmented, size-selected, end-repaired, A-tailed, and ligated to sequencing adapters. DNA nanoballs were generated by rolling-circle amplification, and the libraries were sequenced using an MGISEQ-series sequencing platform at BGI (Qingdao, China).

Raw sequencing reads were filtered using SOAPnuke v2.1.5 [52]. Reads were removed when ≥50% of their sequence matched adapter sequences, when bases with quality scores < 12 accounted for ≥50% of the read length, or when ambiguous bases accounted for ≥10% of the read length.

Clean reads were aligned to the *T. ovatus* reference genome (GenBank accession no. GCA_900607315.1) using Sentieon v201711.03 [53], which incorporates an optimized implementation of BWA-MEM [R4]. Alignments were sorted according to genomic coordinates, and PCR- and optical-duplicate reads were marked.

SNPs were called using the HaplotypeCaller workflow implemented in Sentieon v201711.03. Variant calling was first performed separately for each sample in genomic VCF format, followed by joint genotyping to generate a population-level SNP dataset. SNPs were excluded when QD < 2.0, FS > 60.0, MQRankSum < −12.5, ReadPosRankSum < −8.0, or SOR > 3.0. SNPs with genotype call rates ≤ 60% were additionally removed.

### 4.7. Genetic Diversity and Population Differentiation

Genetic diversity was evaluated separately for the five populations using the quality-controlled Changxin-I genotype dataset. Observed heterozygosity (*H*o) and expected heterozygosity (*H*e) were calculated using VCFtools v0.1.16 [54] with the --het option. For each individual, *H*o and *H*e were calculated as:

$$Ho\;=\;1\;-\;\frac{0(\mathrm{HOM})}{N}$$

and

$$He=1-\frac{e(\mathrm{HOM})}{N}$$

where O(HOM) and E(HOM) are the observed and expected numbers of homozygous loci, respectively, and N is the number of non-missing SNP loci. Population-level *H*o and *H*e were obtained by averaging the corresponding individual estimates.

Allele frequencies were calculated for each population using the --freq option in VCFtools. Polymorphism information content (PIC) was then calculated for each biallelic SNP according to:

PIC = 1 − (*p*^2^ + *q*^2^) − 2*p*^2^*q*^2^

where *p* and q are the frequencies of the two alleles. Mean PIC was calculated across SNPs for each population.

Nucleotide diversity (π) was estimated using VCFtools with a 100 kb sliding window and a 10 kb step (--window-pi 100,000 --window-pi-step 10,000). Mean π across windows containing SNPs was used to characterize nucleotide diversity within each population. Because these estimates were obtained from the predefined Changxin-I SNP markers rather than from all genome-wide variants, they were treated as nucleotide diversity estimates based on the SNP panel.

Hardy–Weinberg equilibrium (HWE) was tested separately for each SNP within each population using the --hardy option in VCFtools. A nominal *p* < 0.05 was considered evidence of deviation from HWE. To account for multiple testing, HWE *p*-values were further adjusted within each population using the Benjamini–Hochberg procedure [55], with an FDR-adjusted *p* < 0.05 considered statistically significant. The adjustment was performed in R using *p* adjust with method = “BH”.

Genetic differentiation between pairs of populations was estimated using the Weir and Cockerham *F*_ST_ estimator implemented in VCFtools [56]. Pairwise comparisons were performed among all five populations using the --weir-fst-pop option, and weighted *F*_ST_ values were used to describe population differentiation. The statistical significance of pairwise *F*_ST_ estimates was assessed using a permutation test implemented in a custom Python script. For each population pair, individual population labels were randomly permuted 1999 times while preserving the original sample sizes, and the weighted Weir–Cockerham *F*_ST_ was recalculated for each permutation. Empirical *p*-values were calculated as (b + 1)/(B + 1), where b represents the number of permuted *F*_ST_ estimates greater than or equal to the observed value and B is the total number of permutations. The resulting *p*-values for the ten pairwise comparisons were adjusted using the Benjamini–Hochberg false discovery rate (FDR) procedure, with an FDR-adjusted *p*-value below 0.05 considered statistically significant.

Nei’s standard genetic distance was additionally calculated from population allele frequencies [57]. For two populations, genetic identity (I) was calculated as:

$$I\;=\;\frac{\Sigma_{i}\;(p_{1i}p_{2i}\;+\;q_{1i}q_{2i})}{\surd [\Sigma_{i}\;({p_{1i}}^{2}\;+\;{q_{1i}}^{2})\;\Sigma_{i}\;({p_{2i}}^{2}\;+\;{q_{2i}}^{2})]}$$

and Nei’s genetic distance was calculated as:

*D* = −*ln*(*I*)

where p_1i_ and q_1i_ denote the frequencies of the two alleles at locus i in the first population, and p_2i_ and q_2i_ denote the corresponding allele frequencies in the second population.

### 4.8. Population Structure Analysis and WGS-Based Validation

Population structure was analyzed independently using the Changxin-I and WGS SNP datasets from the same 144 individuals. The two SNP datasets were analyzed separately using the same population assignments and analytical framework and were not combined into a single genotype dataset.

Principal component analysis (PCA) was performed using PLINK. Principal-component coordinates and eigenvalues were extracted from the PLINK outputs, and the percentage of genetic variation represented by each principal component was calculated from the corresponding eigenvalues. The PCA results were visualized according to the five predefined populations. The PCA files used in the present analysis were standard PLINK pca.eigenvec and pca.eigenval outputs.

Population genetic structure was further analyzed using ADMIXTURE v1.3.0 [58]. Values of K = 2–5 were used to examine changes in genetic structure at different assumed numbers of genetic clusters. For each K, individuals were maintained in the same order and arranged according to the predefined populations ZJ, BH, SY, SZ, and LS. ADMIXTURE Q matrices were visualized as stacked bar plots, with each individual represented by a single vertical bar and different colors indicating the inferred genetic components.

Phylogenetic relationships among individuals were inferred from the filtered SNP datasets using maximum-likelihood analysis implemented in IQ-TREE v2.2.2.6 [59]. The resulting Newick trees were visualized using the Interactive Tree Of Life (iTOL) [60] in a circular layout, with population membership indicated by an outer annotation strip. The trees were treated as unrooted representations of genetic relationships because no external outgroup was included. Phylogenetic relationships among individuals were inferred using maximum-likelihood analysis implemented in IQ-TREE v2.2.2.6.

## 5. Conclusions

In this study, we developed Changxin-I, a 40K liquid SNP array for *T. ovatus*, containing genome-wide and trait-associated markers with high genotyping performance. The array effectively captured the major patterns of genetic diversity and population structure observed in WGS analyses. The SNP array provides a practical genomic resource for population genetics, germplasm management, and future genomic breeding applications in golden pompano.

## Figures and Tables

**Table 1:** Genetic diversity parameters of five cultured *T. ovatus* populations based on Changxin-I genotypes.

Population	*H*o	*H*e	PIC	*π* (×10^−5^)	HWE
ZJ	0.4678	0.4629	0.3498	5.8331	NS
BH	0.4604	0.4705	0.3545	5.9318	NS
SY	0.4718	0.4529	0.3439	5.7114	NS
SZ	0.4776	0.4555	0.3453	5.7439	NS
LS	0.4627	0.4676	0.3521	5.8953	NS

**Note:** N, number of individuals; *H*o, observed heterozygosity; *H*e, expected heterozygosity; PIC, polymorphism information content; *π*, nucleotide diversity; HWE, Hardy–Weinberg equilibrium; NS, no SNP remained significantly deviated from HWE after Benjamini–Hochberg false discovery rate correction. Genetic diversity estimates were based on the 40,398 SNPs retained after quality control.

**Table 2:** Pairwise genetic differentiation *F*_ST_ and Nei’s genetic distance among five cultured *T. ovatus* populations based on Changxin-I genotypes.

Population	ZJ	BH	SY	SZ	LS
ZJ	—	0.0227	0.0639	0.0378	0.0297
BH	0.0090	—	0.0535	0.0354	0.0258
SY	0.0547	0.0433	—	0.0764	0.0671
SZ	0.0269	0.0237	0.0685	—	0.0302
LS	0.0157	0.0109	0.0564	0.0171	—

**Note:** Pairwise *F*_ST_ values are shown below the diagonal, and Nei’s genetic distances are shown above the diagonal. All pairwise *F*_ST_ estimates were statistically significant based on 1999 permutations (empirical *p* = 0.0005; Benjamini–Hochberg FDR-adjusted q = 0.0005 for all ten comparisons). Statistical significance was evaluated at an FDR threshold of 0.05.

**Table 3:** Information on *T. ovatus* samples. Population names correspond to their collection sites, comprising ZJ, BH, SY, SZ and LS.

Location	Abbreviation	Sample Size
Zhanjiang	ZJ	29
Beihai	BH	30
Sanya	SY	30
Shenzhen	SZ	29
Lingshui	LS	26

**Table 4:** Composition of SNP markers included in the Changxin-I 40K liquid SNP array for *T. ovatus*.

Marker Category	Number of SNPs
Genome-wide background SNPs	40,442
Growth-related SNPs	21
Hypoxia-tolerance-related SNPs	5
Cryptocaryon irritans-resistance-related SNPs	5
Sex-associated SNPs	3
**Total**	40,476

## Data Availability

The whole-genome resequencing data generated in this study have been deposited in the National Center for Biotechnology Information (NCBI) under BioProject accession number PRJNA1041556.
